# High-power radiofrequency ablation with optimized pulsing algorithm for renal tumors: initial clinical experience and safety profile

**DOI:** 10.1186/s41747-026-00833-9

**Published:** 2026-11-06

**Authors:** Giovanni Mauri, Eleonora Rossi, Pierluigi Glielmo, Marco Solbiati, Eric Cosman, Luca Maria Sconfienza

**Affiliations:** 1 Unit of Diagnostic and Interventional Radiology, IRCCS Ospedale Galeazzi-Sant’Ambrogio, Milan, Italy; 2 Dipartimento di Scienze Biomediche per la Salute, Università degli Studi di Milano, Milan, Italy; 3 Università degli Studi di Milano, Milan, Italy; 4 Cambridge Interventional LLC, Burlington, MA USA

**Keywords:** Carcinoma (renal cell), Electric impedance, Feasibility studies, Kidney neoplasms, Radiofrequency ablation

## Abstract

**Abstract:**

Incidental diagnosis of renal cell carcinoma is increasing. While surgery remains the standard treatment for localized T1b or larger lesions, image-guided percutaneous ablation represents a valid alternative for T1a cases. Radiofrequency ablation (RFA) is effective but limited by smaller ablation volumes and sensitivity to the heat-sink effect. Microwave ablation and cryoablation have emerged to address these limitations, although the first is susceptible to thermal injury to adjacent structures and the second requires multiple, larger probes and longer procedures. High-power RFA (HP-RFA) is a recent development that delivers higher energy (370 *versus* 200 W), enabling faster and larger ablations. This study evaluated the feasibility and safety of a novel HP-RFA system incorporating an impedance-controlled pulsed algorithm and internally cooled electrodes. Five patients with small renal tumors (mean diameter 2.3 cm) unsuitable for surgery underwent ultrasound- or computed tomography-guided HP-RFA. Technical success, safety, and early tumor control were assessed. All procedures were technically successful. No major complications occurred; one minor perirenal hematoma was managed conservatively. Follow-up imaging at 6 months demonstrated complete tumor ablation without local recurrence. These preliminary findings suggest that HP-RFA is feasible and safe for treating small renal tumors and may represent a promising evolution of RFA. Further studies are needed.

**Key Points:**

- ***Question*** Can HP-RFA provide feasible and safe minimally invasive treatment for small renal tumors while overcoming limitations of conventional RFA?
- ***Findings*** Technical success and efficacy of HP-RFA were achieved in 100% of cases. No major complications. Complete tumor ablation was accomplished.
- ***Relevance statement*** HP-RFA demonstrates feasibility, safety, and effective early tumor control in small renal tumors. By enabling larger, faster ablations, it may overcome limitations of conventional RFA and expand the employment of minimally invasive treatment options instead of surgical approaches.

**Graphical Abstract:**

**Figure:**
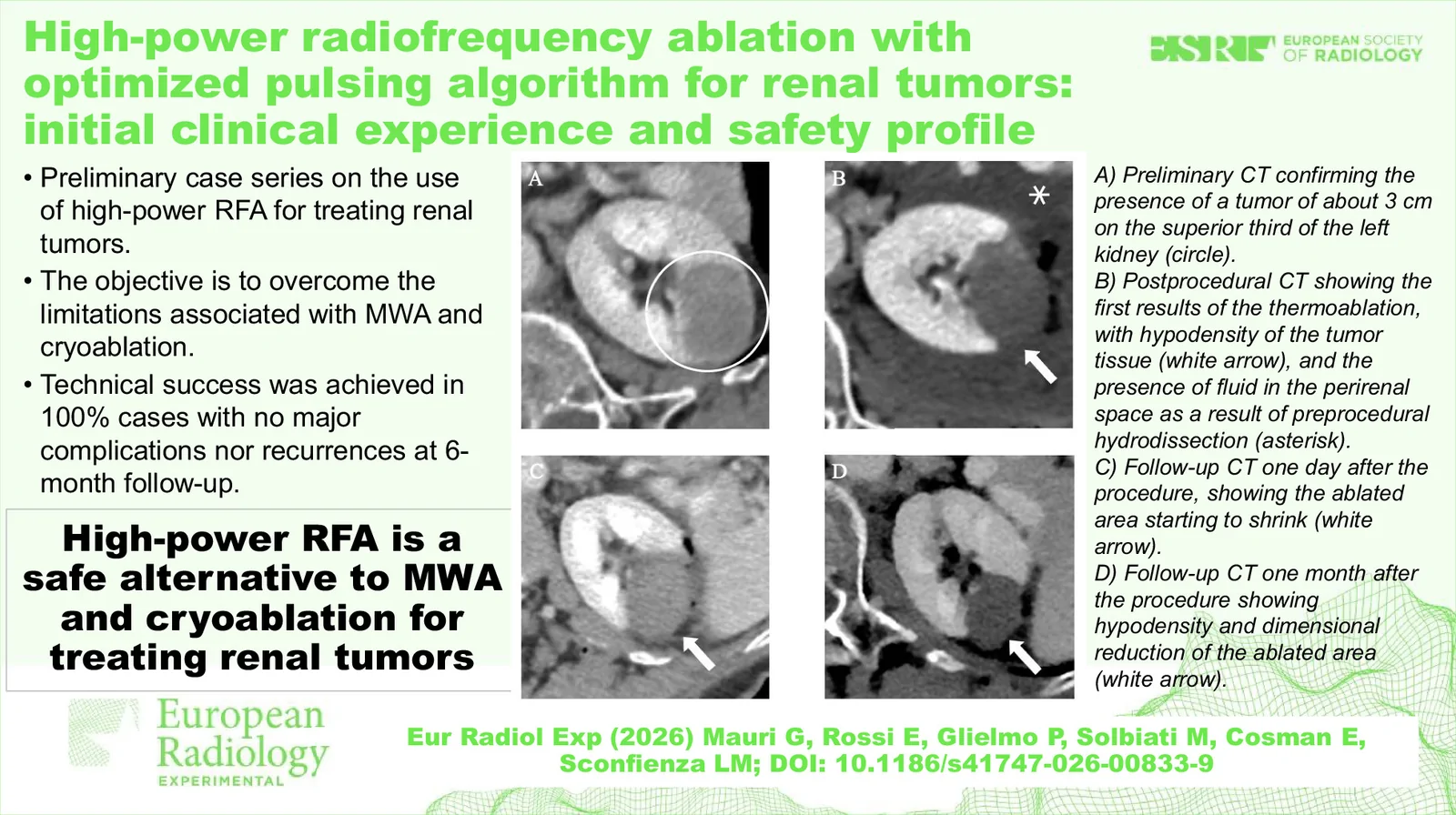

## Introduction

Renal cell carcinoma (RCC) accounts for approximately 2% of all malignancies, and its incidental diagnosis is progressively increasing due to the widespread use of cross-sectional imaging [1–4]. In patients with localized disease, nephron-sparing surgery remains the standard of care; however, image-guided percutaneous ablation has become an established alternative for selected patients, particularly those unfit for surgery or at risk of renal function impairment [2–4]. As a matter of fact, minimally invasive ablative techniques have already proven to be equally effective for treating stage T1a tumors, with comparable or fewer complications related to the procedure [3–5].

Radiofrequency ablation (RFA) was the first percutaneous technique to be widely adopted for the treatment of small renal masses and it still represents the reference standard due to its reproducibility and favorable safety profile [6, 7]. Nevertheless, its clinical use has traditionally been limited to smaller lesions, as conventional systems may be affected by the heat-sink effect and by the limited volumes of necrosis achievable [6, 8, 9].

Microwave ablation (MWA) has recently emerged as an alternative approach, particularly for larger or more vascularized tumors, due to its ability to generate higher temperatures and larger ablation zones with reduced sensitivity to perfusion-mediated cooling [10, 11]. However, the higher energy delivery and thermal spread may also be associated with an increased risk of damage to adjacent structures, including the collecting system, where excessive heating may result in urine overheating and subsequent ureteral injury, with the frequent need to prevent this damage with techniques such as pyeloperfusion [11, 12]. Cryoablation has also emerged as an alternative, hypothermic modality because it can create large volumes of ablation with a low rate of injury to the renal collecting system. However, cryoablation requires the use of multiple, usually thicker probes, and longer treatment times, and the necrotic changes within the volumes of ablation may be inhomogeneously distributed [12].

Recent technological advancements have led to the development of a high-power RFA (HP-RFA) system that nearly doubles the maximum power of traditional RFA systems (370 *versus* 200 W) and incorporates optimized pulsing algorithms and internally cooled electrodes with longer maximum active tip lengths (5 *versus* 3 cm), designed to increase energy delivery while maintaining controlled thermal deposition [13].

The aim of this study was to evaluate the feasibility and safety of a new-generation HP-RFA system for the treatment of small renal tumors, providing preliminary clinical data on this emerging technology.

## Materials and methods

For this retrospective study, Institutional Review Board approval from Comitato Etico Territoriale Lombardia 1 (protocol RETRORAD v6, emend 12/01/2026) was obtained. The Ethics Committee granted a waiver for informed consent.

Five patients (3 males, 2 females) with a mean age of 69.2 years (range 60–76) with a biopsy-proven RCC (4 clear cell RCC, 1 papillary RCC) with a mean maximum diameter of 2.3 cm (range 1–3 cm) and considered unsuitable for surgery or at high surgical risk after multidisciplinary evaluation because of comorbidities were treated with HP-RFA. Tumor anatomical complexity was assessed using the Preoperative Aspects and Dimensions Used for an Anatomical (PADUA) classification [14].

All procedures were performed in a dedicated interventional suite equipped with ultrasound and computed tomography (CT). The position of the patients was accurately planned in order to allow the most direct access to the target lesion. Procedures were carried out by a team of two interventional radiologists, including at least one with more than 10 years of experience.

Adjunctive techniques, such as hydrodissection, were selectively employed based on lesion location and proximity to critical structures (more specifically, lung or bowel) [9, 11]. A preliminary ultrasound evaluation was performed to plan the needle trajectory and patient positioning, followed by contrast-enhanced CT (CECT) to better define tumor characteristics and the surrounding anatomy.

The electrode was advanced under ultrasound or combined ultrasound/CT fusion guidance, with additional CT acquisitions performed to confirm correct positioning [15]. Ablation was conducted at a single location, using a single probe, using preset generator settings for the applicable electrode active tip length (2, 3, or 4 cm) using an impedance-controlled pulsed algorithm, with a mean duration of 12 min according to protocol.

The procedures were all performed under general anesthesia.

At the end of the procedure, CECT was performed to assess treatment completeness and detect immediate complications. Patients were observed overnight and discharged the following day if clinically stable.

Imaging follow-up with CT was performed at 1 day, 1 month, and 6 months after treatment.

### Definitions and endpoints

Technique efficacy was defined as complete coverage of the target lesion by the ablation zone on immediate post-procedural imaging.

Primary endpoints were feasibility (technique efficacy) and safety (complications classified according to Society of Interventional Radiology criteria [16]).

The secondary endpoint was early local tumor control, defined as the absence of contrast-enhancing tumor tissue within or at the margin of the ablation zone during follow-up.

### HP-RFA system

An HP-RFA system (Cambridge Interventional CRF System) was used in all procedures. This system includes a generator capable of delivering up to 370 W and 2,500 mA output at 50 Ω, a fluid circulation unit, an internally cooled electrode system consisting of a 17-gauge electrode, and grounding pads [17].

The generator employs an impedance-controlled pulsed algorithm to optimize energy deposition. After an initial ramp-up phase (approximately 30 s) to reach a target current based on electrode tip length, energy delivery is dynamically modulated according to real-time tissue impedance [17]. During stable impedance, current is increased in 100 mA increments at 30-s intervals [13, 17]. When impedance rises, energy delivery is temporarily interrupted (≥ 20 s) and subsequently resumed; if impedance increases rapidly, the current is reduced by 100 mA before continuation [13, 17].

For each ablation, the active tip length of the cannula (2, 3, or 4 cm) was selected according to lesion characteristics. Also, continuous circulation of chilled saline solution (~4 °C) was employed to maintain the electrode temperature ≤ 22 °C, limiting tissue charring and enabling higher current delivery [13, 17].

This combination of high power and current output, impedance-modulated pulsed delivery, and internal cooling is designed to enhance energy transmission, allowing the creation of larger and more homogeneous ablation zones while maintaining a controlled thermal profile [13, 17].

## Results

Technical success and technique efficacy were achieved in all cases (5/5, 100%). All patients tolerated the procedure well and were discharged the day following the ablation.

No major complications were observed (0/5, 0%). One patient of the 5 patients (1/5, 20%) developed a minor complication, developing a small perirenal hematoma without active bleeding. The hematoma was managed conservatively with manual compression, with no clinical sequelae.

Follow-up imaging demonstrated complete ablation in all treated lesions, with no evidence of local tumor progression at 6 months.

The size range of the ablation volumes ranged from 15.3 to 38.5 mm (mean: 28.8 mm).

The characteristics of the patients and the results of the procedures are summarized in Table 1.

**Table 1:** Summary of the characteristics of the patients and the results of the procedures

Patients	Gender	Age (years)	Tumor size (cm)	PADUA Score	Imaging guidance	Active tip (cm)	Procedure timing (min)	Ablation timing (min)	Hydro-dissection	Complications	Local recurrences at follow-up
1	Male	70	2.4	7	US/CT fusion imaging	3	102	12	No	None	No
2	Female	60	1.0	6	US-guidance and CT-guidance	3	98	12	No	None	No
3	Male	76	3.0	5	US-guidance and CT-guidance	2	73	12	No	None	No
4	Female	66	3.0	7	CT-guidance	2	68	12	Yes	None	No
5	Male	74	2.0	2	US-guidance and CT-guidance	2	99	12	Yes	Small local hematoma	No

*CT* Computed tomography, *PADUA* Preoperative aspects and dimensions used for an anatomical classification (see ref. [15]) *US* Ultrasound

**Fig. 1:**
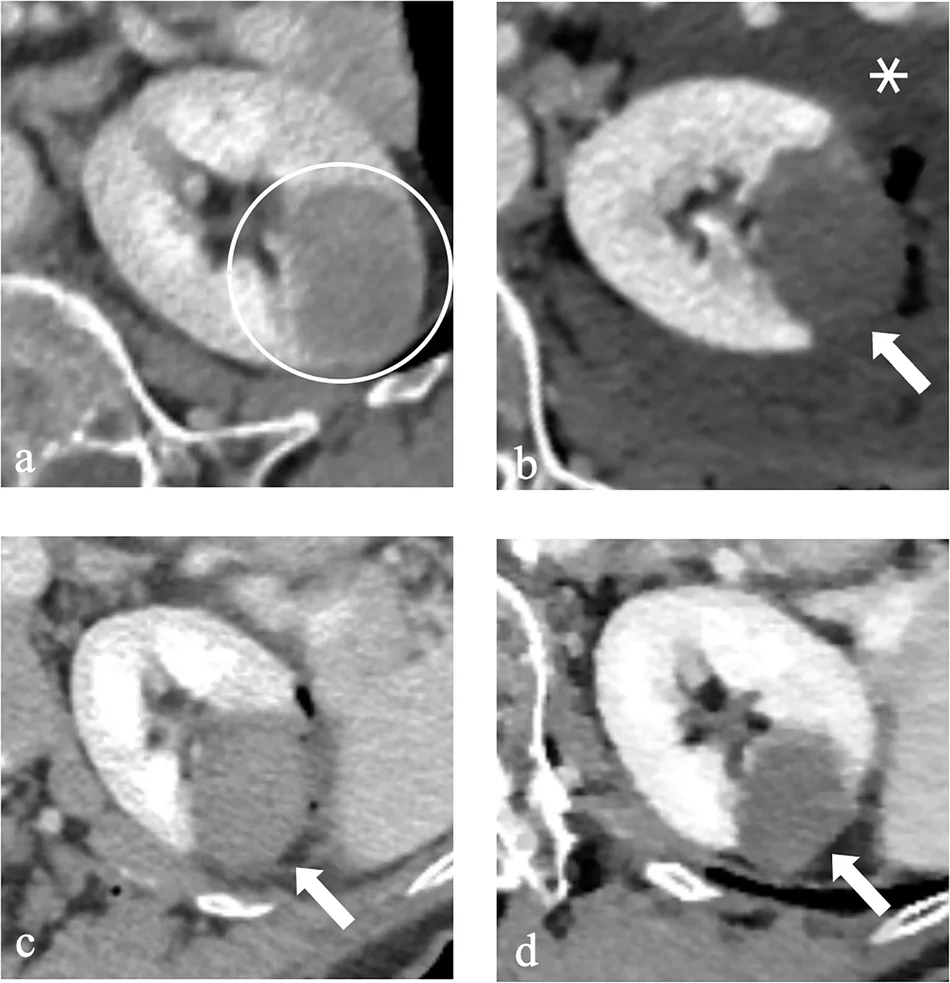
Images showing preprocedural and postprocedural CT-scan assessments and follow-up images after one day and after one month from HP-RFA. **a** Preliminary CT scan confirming the presence of a tumor of about 3 cm on the cortex of the superior third of the left kidney (circle). **b** Postprocedural CT scan showing the first results of the thermoablation, evidenced by hypodensity of the tumor tissue (white arrow), and the presence of fluid in the perirenal space as a result of preprocedural hydrodissection (asterisk). **c** Follow-up CT scan one day after the procedure, showing the ablated area starting to shrink (white arrow). **d** Follow-up CT scan one month after the procedure showing hypodensity and dimensional reduction of the ablated area (white arrow). CT, Computed tomography

**Fig. 2:**
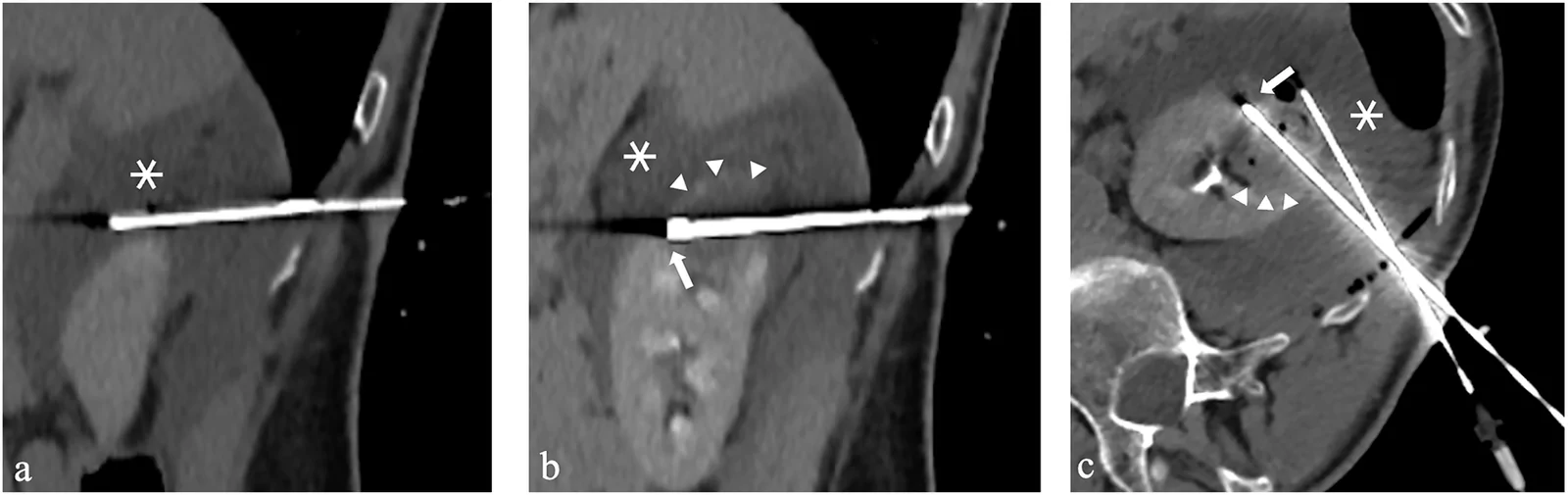
Images showing the placement of the hydrodissection needle and the probe. **a** Multiplanar reconstruction of the CT image showing the correct placement of the hydrodissection needle and the fluid injected in the perirenal space (asterisk). **b** Multiplanar reconstruction of the CT image showing the renal lesion (whose superior margins are marked with three arrowheads) with the probe correctly positioned in the center of the lesion (white arrow) and the hydrodissection fluid in the perirenal space (asterisk). **c** Axial CT image showing the probe localized at the center of the renal lesion (white arrow), with the medial margins of the tumor marked by the three arrowheads; the hydrodissection needle is positioned in proximity to the lateral margin of the renal lesion (circle); it is also possible to appreciate the results of the hydrodissection in the perirenal space (asterisk). CT, Computed tomography

**Fig. 3:**
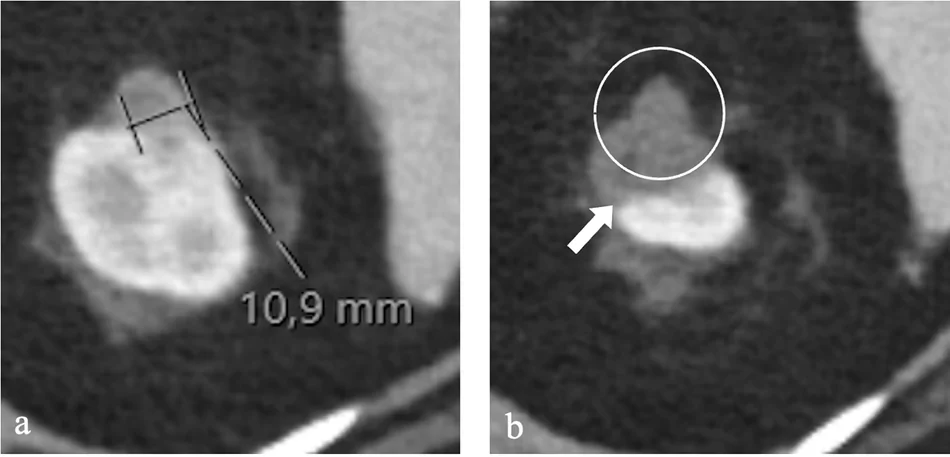
Images showing the renal tumor before and after ablation. **a** Preliminary CT scan showing a small renal tumor (about 11 mm) with partially exophytic extension at the superior pole of the left kidney. **b** Follow-up CT image one month after RFA demonstrates regular results of the thermoablation, demonstrated by hypodensity of the tumor area (circle); it is also possible to appreciate an area of hypodensity in the renal tissue peripheral to the ablated tumor due to ischemic outcomes of RFA (white arrow). CT, Computed tomography; RFA, Radiofrequency ablation

The case of one patient treated with HP-RFA is shown in Figs. 1 and 2. The CT images demonstrating the results of the procedure after one month are shown in Fig. 3.

## Discussion

In this study, HP-RFA was successfully performed in all patients, with no major complications (only one minor adverse event), and no evidence of local tumor progression at short-term follow-up. Although limited by the small cohort, these findings support the feasibility of the technique and suggest that increased power delivery can be achieved without compromising procedural safety.

From a technical standpoint, the system used in this study combines internally cooled electrodes with an optimized pulsing algorithm, allowing efficient and controlled energy delivery [13, 15]. Previous studies in the liver have demonstrated that this approach can increase ablation size while reducing procedure duration [13]. Our preliminary data represent the first clinical experience with this HP-RFA system for renal tumors on human subjects, suggesting that these advantages may also translate to the renal setting.

Importantly, HP-RFA enabled effective ablation without requiring modifications to standard workflows. Protective maneuvers were only selectively applied, and notably no pyeloperfusion was needed in any case, suggesting that increased power delivery did not result in greater procedural complexity or additional risk.

Although no direct comparison was performed, the standardized 12-min ablation protocol used for HP-RFA is not substantially longer than treatment times typically reported for MWA, suggesting that improved controllability and safety may be achieved without a major time penalty.

The main limitations of our study are its retrospective design, the limited sample size, and the relatively short follow-up period. Therefore, no conclusions can be drawn regarding long-term oncological outcomes or comparative efficacy, and the present findings should be considered hypothesis-generating. Nevertheless, these preliminary results provide an important proof-of-concept supporting the feasibility and safety of HP-RFA for the treatment of renal tumors.

The growing adoption of microwave and cryoablation has contributed to the perception that RFA may progressively lose its role, particularly in more complex lesions. Our preliminary experience suggests that this assumption may be premature. Rather than being superseded, RFA may continue to evolve through technological innovation. HP-RFA may represent a promising strategy to improve ablation performance while maintaining the intrinsic safety and controllability of radiofrequency energy. By leveraging technological advances already validated in hepatic applications, this approach could potentially expand the indications of RFA and contribute to redefining the balance between efficacy and safety among percutaneous ablative techniques.

## Conclusion

Further prospective studies with larger cohorts, longer follow-up, and direct comparison with other ablative modalities are warranted to better define the clinical role of HP-RFA and to confirm these preliminary observations.

## Data Availability

The datasets used and/or analyzed during the current study are available from the corresponding author on reasonable request.

## Abbreviations

CECT: Contrast-enhanced computed tomography
CT: Computed tomography
HP-RFA: High-power radiofrequency ablation
MWA: Microwave ablation
RCC: Renal cell carcinoma
